# Components reuse in the building sector – A systematic review

**DOI:** 10.1177/0734242X20910463

**Published:** 2020-02-29

**Authors:** Kambiz Rakhshan, Jean-Claude Morel, Hafiz Alaka, Rabia Charef

**Affiliations:** 1 Centre for Research in the Built and Natural Environment, Coventry University, UK; 2 Digital Technologies Lab, University of Hertfordshire, UK

**Keywords:** Reuse, building components, systematic literature review, building sector, construction and demolition waste, circular economy, superstructure

## Abstract

Widespread reuse of building components can promote the circularity of materials in the building sector. However, the reuse of building components is not yet a mainstream practise. Although there have been several studies on the factors affecting the reuse of building components, there is no single study that has tried to harmonize the circumstances affecting this intervention. Through a systematic literature review targeting peer-reviewed journal articles, this study intends to identify and stratify factors affecting the reuse of components of the superstructure of a building and eventually delineate correlations between these factors. Factors identified throughout this study are classified into six major categories and 23 sub-categories. Then the inter-dependencies between the barriers are studied by developing the correlation indices between the sub-categories. Results indicate that addressing the economic, social and regulatory barriers should be prioritized. Although the impact of barriers under perception, risk, compliance and market sub-categories are very pronounced, the highest inter-dependency among the sub-categories is found between perception and risk. It suggests that the perception of the stakeholders about building components reuse is affected by the potential risks associated with this intervention.

## Introduction

The construction industry consumes between 30% and 50% of the natural resources (Anink et al., 1996; Herczeg et al., 2014; World Steel Association, 2012), produces up to 40% of the total waste stream (excluding the excavation waste) (Clark et al., 2006; Defra, 2019; Eurostat, 2019; UNEP, 2015) and generates around 39% of the world’s greenhouse gas emissions (Abergel et al., 2017). The above facts are alarming due to the urgent need to decrease greenhouse gases (GHG) (United Nations Framework Convention on Climate Change, 2015) and because we are facing landfilling restrictions (Brewer and Mooney, 2008) and resource deficiency globally (Chen et al., 2010; Ellen MacArthur Foundation, 2013).

The depletion of the earth’s resources as the result of fast economic expansion, continuous population growth and the drastic increase in demand for products and services has led governments to run resource-efficient economies (Ellen MacArthur Foundation, 2013). Therefore, the regulatory authorities worldwide, such as the European Commission Waste Framework Directive 2008/98/EC (EU, 2008) and the Demolition Protocol (ICE, 2008), introduce waste hierarchies to improve the material efficiency across all the economic sectors, including the building industry. According to these waste hierarchies, ‘preparing for re-use’ (or simply ‘reuse’) is the second-best solution after ‘prevention’ to decrease the high level of waste generation and to decouple economic growth from resource consumption.

New design methods such as design for deconstruction (DfD) (Akinade et al., 2017; Tingley and Davison, 2011) and design for manufacture and assembly (DfMA) (Kalyun and Wodajo, 2012) have been introduced to prevent or decrease waste throughout the entire lifecycle of new buildings. However, most of the existing buildings are not designed based on the above techniques, which results in the generation of a considerable amount of wastes during refurbishment or the demolition phase. Although, according to the waste hierarchies, reuse is preferred to recycling, most of the recovery of construction and demolition wastes (CDW) happens in the form of recycling and not reuse. For example, in the UK, nearly 91% of the non-hazardous CDW is recovered through recycling (Defra, 2019).

Although recycling can divert waste from the landfills, the processes involved are energy and resource-intensive and impose a noticeable pressure on the environment in terms of GHG and other sorts of emissions (Addis, 2006; WRAP, 2008). Contrarily, reused building components (bricks, beams, columns, truss, etc.) have far lower environmental impacts when compared with recycled materials (Geyer et al., 2002). For instance, when new steel sections which consist of around 60% recycled content are used, their environmental impacts are still 25 times higher than reusing the equivalent reclaimed steel sections (WRAP, 2008). According to Lazarus (2003), reusing reclaimed structural steel or timber sections can decrease the environmental impacts by 96% and 83%, respectively. It is primarily due to the significantly lower treatment and reprocessing required for building components reuse (BCR) in comparison with recycling (Gorgolewski et al., 2008). Notwithstanding, the reuse rates in the building sector have declined in the last two decades in countries like the UK (Addis, 2006; Sansom and Avery, 2014), and only a fraction of components at the end-of-life of a building are reused (e.g. the 5% reuse rate for the reclaimed steel sections in the UK in 2012 (Sansom and Avery, 2014)).

Building components reuse, and the factors affecting its uptake has been the focus of research for several years. However, there is no evaluation material synthesizing the factors affecting BCR to find the correlations between these factors and harmonize the circumstances affecting the reuse of building components. In the lack of such a study, reuse will not grow in the building industry because the fragmented body of knowledge available in the literature is unable to direct the stakeholders to take progressive steps towards circularity of materials in this sector. This study thus aims to bridge this gap by analysing different aspects that influence the adoption of component reuse in new buildings, prioritizing the barriers to reuse in terms of their urgency to tackle and draws roadmaps for future research. The authors intend to achieve these goals through a systematic review approach targeting peer-reviewed journal articles. Therefore, the authors identify the following objectives for the aim of this study:

(i) To identify and stratify drivers and barriers affecting components reuse in the building sector.
(ii) To delineate correlations between the barriers to prioritize the necessary actions.

### Definitions, scope and limitations

The following terms are used frequently in this study and are defined as follows:

*Adaptive reuse/building reuse*. Extending the life of an entire building (or at least some parts of it, for example, its structure) at the end of its useful life due to its historical/social values (Addis, 2006; Gorgolewski, 2008).
*Deconstruction*. Careful disassembly of a building to maximize the reusability of its constituents (Addis, 2006; Munroe et al., 2006).
*Recycling*. A set of steps to collect, sort, transport, process and convert a discarded material (scrap metal, packaging cartons, concrete blocks, etc.) into new products (new steel plates, recycled papers, recycled concrete aggregate (RCA), etc.) (Ferrer and Clay Whybark, 2000).
*Building material reuse (BMR)*. The use of building materials (e.g. RCA, crushed bricks, etc.) in the production of new building components (concrete columns, slabs, beams, etc.) (Addis, 2006).
*BCR*. Bringing back a discarded building component (e.g. a beam, column, bricks, windows, doors, etc.) into its original function with minimum (or zero) treatments (Addis, 2006; Parker and Deegan, 2007).
*Reverse logistics*. A set of interventions (e.g. recycling, reuse, etc.) or design strategies (DfD, DfMA, etc.) to minimize CDW during the entire life cycle of a building (Aidonis et al., 2008; Hosseini et al., 2015; Iacovidou and Purnell, 2016).

The scope of this study is limited to peer-reviewed journal articles because these types of research works are considered of high quality and validity (Schlosser, 2007). This approach is in line with Yi and Chan’s (2014) advice to investigate top-tier construction journals while performing literature reviews.

This paper focuses on BCR and other types of reuse, such as adaptive reuse, recycling and BMR, are out of the scope of this study. Although adaptive reuse is the most preferred option to prevent waste, because this paper focuses on the management of CDW after generation, adaptive reuse is out of the scope of this review. As explained in the introduction section, other waste treatment options such as recycling and BMR are highly energy and resource-intensive (Addis, 2006; WRAP, 2008) and are therefore not considered to be in the scope of this study. This trend is followed while selecting the proper search words in the methodology section as well.

The terms building component(s) and element(s) are used interchangeably in this study. These are restricted to sections forming the superstructure of a building as defined by BCIS (2012) that can be dismantled (through demolition, deconstruction or selective demolition) and reused for the same function with minimum (or zero) treatments (Addis, 2006; Parker and Deegan, 2007). Therefore, this study does not consider substructure (foundation), plinth, finishes, fittings, furnishings, equipment and services in its scope (BCIS, 2012).

Two major examinations are performed to scrutinize the articles reviewed in this paper. The first method is focused on identifying and analysing reuse drivers and barriers (cumulatively called factors), and the second method is focused on correlations and the possible inter-relationships between reuse barriers.

The next section explains the methodology employed in this study. The results and discussions section deals with the findings and deeply investigates the identified factors and summarises the study by presenting the discussion and the next steps through recommendations. Eventually, the article presents the conclusions and highlights its contribution to the body of knowledge.

## Methodology

This study uses a systematic literature review method to identify various factors (drivers and barriers) affecting the reuse of building components on a global scale. A systematic review is a comprehensive and reliable process for locating the existing body of knowledge (published scientific work) regarding a very particular research question (GET-IT Glossary, n.d.; Denyer and Tranfield, 2009). Because this process is based on a defined search strategy with clearly specified objective(s), it can be used to analyse, synthesize and critically evaluate the existing literature identified within the context of the research question (Bettany-Saltikov, 2016; Denyer and Tranfield, 2009). This methodology provides a strong basis for reliable judgments about ‘what works’ the best (Petrosino and Lavenberg, 2007) and finds gaps in the literature for further research (Denyer and Tranfield, 2009). The systematic literature review is a well-known methodology for the study of the existing knowledge in medical sciences because of its unique properties, as expressed above (Tranfield et al., 2003). Furthermore, it is also acquiring its position among other research areas such as engineering and management (Alaka et al., 2018, 2016; Charef et al., 2018; Hosseini et al., 2015).

**Figure 1.**
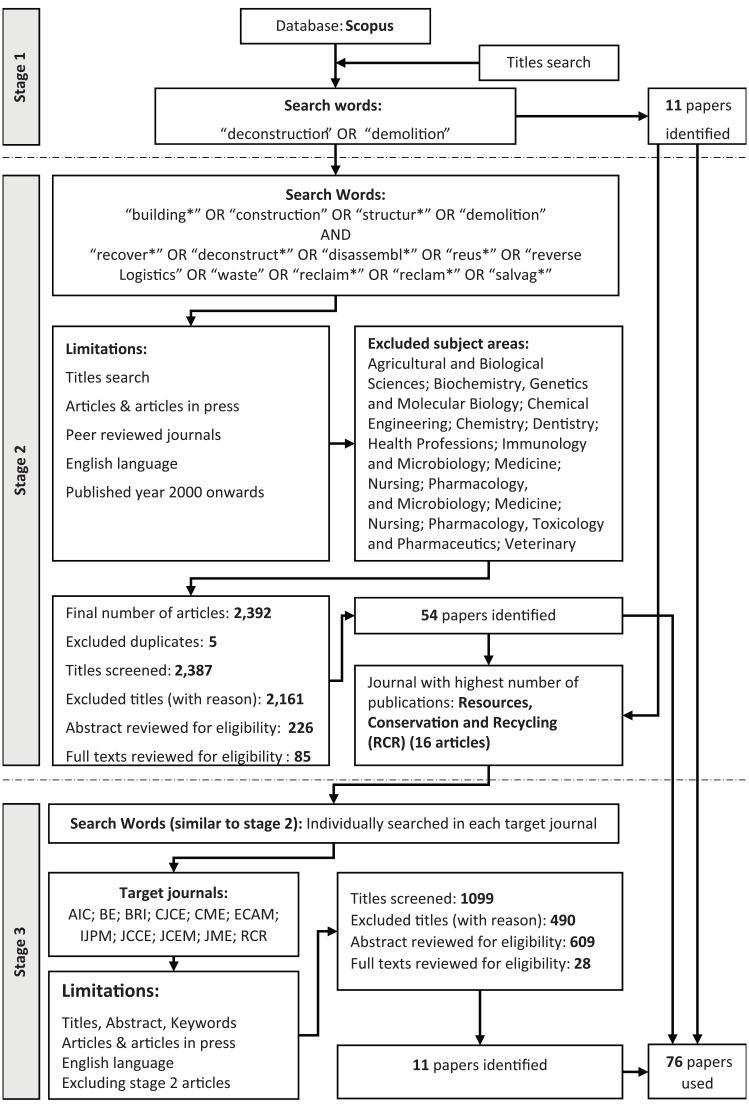
Systematic literature review framework (inspired by Charef et al. (2018), PRISMA (2018) and Yi and Chan (2014)).

The complete process of the systematic literature review is presented in Figure 1. In this study, the Preferred Reporting Items for Systematic Reviews and Meta-Analyses (PRISMA) (PRISMA, 2018) checklist is used to step-by-step perform and record the methodology. The PRISMA checklist is widely used by researchers when performing systematic literature reviews (Moher et al., 2009).

A pre-requisite to conducting a systematic review is a clear research question as well as knowing the proper keywords to perform an effective search. Because a building at the end of its lifecycle is removed through demolition (with some other variations such as selective demolition and deconstruction), to identify the proper keywords, the authors performed an initial literature search using ‘deconstruction’ and ‘demolition’ search words at stage 1. Through this initial search, 11 relevant papers were identified, which helped in the selection of the search words listed in Figure 1 (stage 2).

At stage 2, a Boolean search criterion is followed to answer the research question of this study. At this stage, the search is limited to the ‘titles’ of the articles. The initial search in Scopus showed that studies containing discussions on the reuse of building components focus on construction and demolition waste management. Therefore, the first set of search words intends to ensure that any article containing these words are considered. The AND combination with the second set of search words guarantees that all relevant articles dealing with reuse in the building sector are included in the search. Because the scope of this paper is BCR and not building reuse or BMR, keywords such as ‘refurbish’ or ‘refurbishment’, which primarily deal with adaptive reuse of existing buildings (particularly historic buildings), or ‘material’, which deals with material reuse, are not included in the search words (Figure 1).

The cut-off date for stages 1 and 2 of the literature review is March 2019, whereas the cut-off date for stage 3 is January 2020. Because this study only focuses on peer-reviewed journal papers, following Yi and Chan (2014), all other types of publications (book chapters, conference papers, trade journals, etc.) are excluded. Hence, only ‘Articles’ and ‘Articles in press’ published in peer-reviewed journals are considered for this study. Likewise, to limit the number of unwanted articles, irrelevant subject areas, as listed in Figure 1 at stage 2, are excluded from the search criterion. This is because search words such as ‘building’, ‘construction’, ‘structure’, ‘reuse’ and ‘recover’ are found in a broad range of scientific publications. Furthermore, since most of the publications in this area are published after 2000, stage 2 considers the range of articles published between 2000 and March 2019.

**Figure 2.**
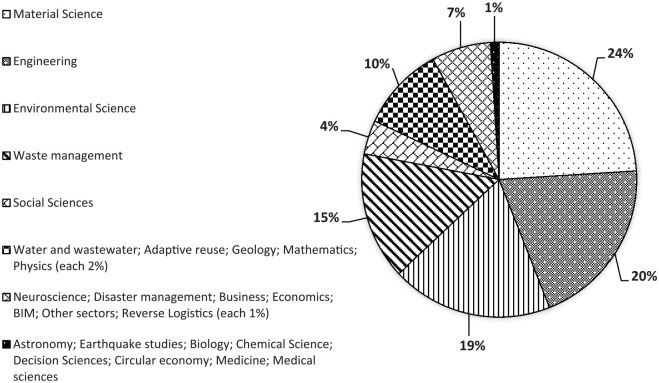
Subject area of the excluded papers during the screening process at stage 2.

Among the 2387 article titles screened at stage 2, 2161 articles were found to be irrelevant and were excluded. Figure 2 depicts the percentage of the subject areas of the excluded papers during the screening stage. The appearance of articles in areas like the medical sciences (which were excluded from the subject areas) could be because of the interdisciplinary nature of some papers. The authors then reviewed the abstracts of the remaining 226 articles during the eligibility check of stage 2 (PRISMA, 2018) (Figure 1). At this stage, irrelevant papers, such as those focusing on construction waste management other than reuse (Guo, 2016; Jin et al., 2017), concentrating on other sectors like reverse logistics in the electronics industry (Sirisawat and Kiatcharoenpol, 2019) or talking about reuse but dealing with recycling or down-cycling (Migliore et al., 2015), were identified and excluded. The result is the exclusion of 141 more papers from the full-text review. The authors eventually reviewed 85 full-text articles from which we could find 54 papers relevant to the objectives of this study.

The search results from stages 1 and 2 indicate that the journal of *Resources, Conservation and Recycling (RCR)* has the highest number of publications (16 papers) among all the reviewed journals. Hence, following the framework pursued by Yi and Chan (2014), a third stage systematic literature review was performed considering all of the 10 first-tier construction journals as well as *Resources, Conservation and Recycling (RCR)*. The complete list of all these journals are *Automation in Construction (AIC); Building and Environment (BE); Building Research and Information (BRI); Canadian Journal of Civil Engineering (CJCE); Construction Management and Economics (CME); Engineering, Construction and Architectural Management (ECAM); International Journal of Project Management (IJPM); Journal of Computing in Civil Engineering (JCCE); Journal of Construction Engineering and Management (JCEM); Journal of Management in Engineering (JME); Resources, Conservation and Recycling (RCR)*. At this stage, the identified search words were used to perform a Boolean search in the ‘title/abstract/keywords’ of each of the journals separately. Moreover, the year 2000 restriction was lifted at this stage (Figure 1). All the above was to overcome the restrictive nature of the stage 2 limitations (Figure 1), as well as to make sure that articles published in high-impact journals related to the built environment were considered.

During this process, 490 articles were excluded from the abstract review for similar reasons observed in stage 2. For instance, while the paper by Ling and Leo (2000) focuses on identifying drivers to promote timber formwork reuse, it is out of the scope of this study, which is the superstructure of a building . After reviewing 609 abstracts during the eligibility check, only 28 papers were identified for a full-text review. Although the reviewed full-texts contained a combination of the search words, the focus of the rejected papers was not in line with the aim of this study. Following the same protocol pursued at stage 2, a total number of 11 more papers were identified at this stage. According to what has been mentioned earlier, and combining the identified papers at all three stages, 76 articles were found to be relevant to the objectives of this paper and were reviewed. Nonetheless, the identified new articles, as a result of the third stage systematic review, were all published after the year 2000, which validates the initial decision in restricting the publication date.

## Results and discussions

**Figure 3.**
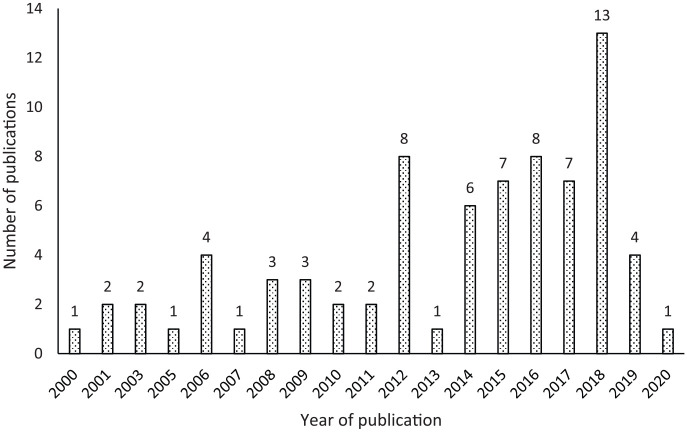
Publications by year.

Figure 3 shows the distribution of the papers reviewed in this study by the year of publication. According to this figure, the number of peer-reviewed journal articles has been increasing since 2014, which indicates an increasing focus on construction and demolition waste treatment through reuse. However, there was a decline in the number of publications in 2019, which needs further investigations to identify the root causes.

**Figure 4.**
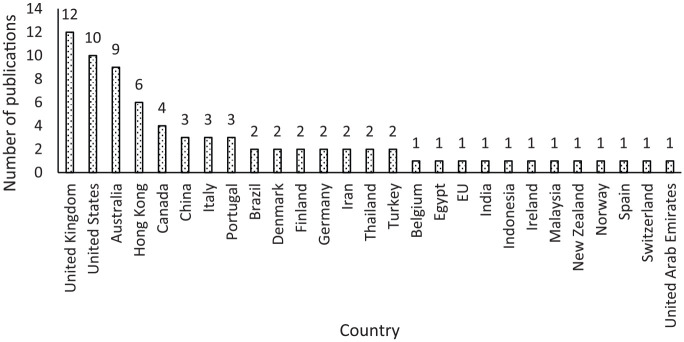
Publications by location.

Figure 4 shows the geographic location of the reviewed articles in this study. According to this figure, waste management in buildings through reuse is an international trend.

Tables 1 and 2 show that the authors of the reviewed papers employed various methodologies to perform their research. These methodologies are identified for the individual papers in Table 1 for reuse drivers and Table 2 for reuse barriers. The variety of techniques used, including various qualitative and quantitative methods, show the attempts made by different authors to study different aspects of BCR, which reveals the increasing importance of this intervention among researchers. For instance, a series of studies performed in Australia employs mixed methodologies such as interviews and questionnaire surveys and targets various stakeholders to investigate drivers and barriers to reverse logistics in the South Australian construction context (Chileshe et al., 2015, 2016a, 2016b, 2018; Rameezdeen et al., 2016). These studies show the importance of a holistic approach in seeking the experts’ opinions (through qualitative methods (Saunders et al., 2016)) and actual experiences (through quantitative methods (Saunders et al., 2016)) to identify deficiencies in the body of knowledge and eventually promote practises like reuse in the building sector. Although it is tempting to discuss different research methods and methodologies employed in the 76 papers reviewed (and compare advantages and limitations of them), the above is out of the scope of this study and can be investigated separately.

**Table 1.** Summary of reuse drivers.

SN	Author	Cntr.^a^	Research method^b^	Categories of reuse drivers^c^																				
				Economic			Env		Organizational					Regulatory			Social					Technical		
				A: Cost	B: Market	C: Value for money	D: Energy and GHG	E: Preservation	F: Contracts	G: Experience	H: Infrastructure	J: Management	K: Sustainability	L: Compliance	M: Incentive	N: Sustainability	O: Awareness	P: Perception	R: Sustainability	S: Trust	T: Willingness	U: Deconstruction	V: Design	X: Information
1	MacKinnon (2000)	US	DR; GI; I(4); OBS	1					1								1							
2	Sára et al. (2001)	IT	CS(1); LR				1	1																
3	Li et al. (2003)	HK	CS(2); S												1									
4	Klang et al. (2003)	US	CS(1); I(10); Q(10/10)	2	1	1	1																	
5	Dantata et al. (2005)	US	CS(5); LR	1		1				1														
6	Pun and Liu (2006)	AU	TF																			1		
7	Pun et al. (2006)	AU	CS(1)			2							1									1		
8	Shaurette (2006)	US	Q(296/83)							1						1				1	1			
9	Guy (2006)	US	CS(4)										1											
10	Schultmann and Sunke (2007)	DE	T										1									1		
11	Gorgolewski et al. (2008)	CA	AR; CS(3)	3					1			1									3	1	2	1
12	Gorgolewski (2008)	CA	AR; CS(2)	3					1			3				1					2		1	1
13	Tam and Tam (2008)	HK	CS(1); I(20)												1		1							
14	da Rocha and Sattler (2009)	BR	CD; CS(1); DO(5); GM(4); SSI(27)	2	1	1														1				
15	Nordby et al. (2009)	NO	CS(1)																					1
16	Dewulf et al. (2009)	BE	CS(1)					1																
17	Denhart (2010)	US	CS(4)			1																1		
18	Rogers (2011)	AE	CS(1)								2	1	2					1			1			
19	Forsythe (2011)	AU	CS(9); DO; UI			1																1		
20	Chau et al. (2012)	HK	CS(13)				1	1																
21	Arif et al. (2012)	IN	CS(2); SSI(15)	1																	1			
22	Lachimpadi et al. (2012)	MY	CS(8)																			1		
23	Boyd et al. (2012)	US	CS(2)				1																	
24	Densley Tingley et al. (2012)	GB	CS(1); LR					1					1									1		
25	Coelho et al. (2012)	PT	CS(15)				1					1												
26	Aye et al. (2012)	AU	CS(1)				1	1					1											
27	Elias Özkan (2012)	TR	AR; CS; DO(21); I							1	1													
28	Hglmeier et al. (2013)	DE	CS(1)																			1		
29	Sansom and Avery (2014)	GB	Q(160/32)																		1			
30	Elias-Ozkan (2014)	TR	CS(2)			1	1	1					1											
31	Pongiglione and Calderini (2014)	IT	AR; CS(1)	1																		1	1	1
32	Durão et al. (2014)	PT	CS(2)										1											
33	Diyamandoglu and Fortuna (2015)	US	CS(1)	1	1	1	1																	
34	Yeung et al. (2015)	CA	DO(4)																			1		
35	Wu et al. (2016)	CN	CA													1								
36	Cooper et al. (2016)	GB	CS(2); LR; SSI(17)	1		1																		
37	Rameezdeen et al. (2016)	AU	SSI(8)																		1			
38	Ding et al. (2016)	CN	CS(1); LR; SSI(12)								1													
39	Chileshe et al. (2016b)	AU	LR; Q(539/49); SSI(6)										2							1	1			
40	Ajayi et al. (2016)	GB	FGI(23)																			1		
41	Chinda and Ammarapala (2016)	TH	CS(2); I(6); LR	1				1					1			1								
42	Chileshe et al. (2016a)	AU	LR; SSI(8)	1									1						1					
43	Tatiya et al. (2017)	US	CS(1); LR; SI(3)	1																				
44	Ajayi et al. (2017)	GB	FS; Q(200/131)								1	1												
45	Surahman et al. (2017)	ID	CS(2)				1						1											
46	Chau et al. (2017)	HK	CS(1)				1																	
47	Dunant et al. (2017)	GB	I(30); Q(24)	1		1															3			
48	Faleschini et al. (2017)	IT	CS(1)				1																	
49	Tingley et al. (2017)	GB	LR; SSI(13)	1	1		1	1		1		2	1								1			
50	Yeung et al. (2017)	CA	CS(1)				1	1																
51	Machado et al. (2018)	BR	LR				1																1	1
52	Gottsche and Kelly (2018)	IE	ACT(1); CS(5)			1	1						1											
53	Gálvez-Martos et al. (2018)	EU	CA										1											
54	Brütting et al. (2019)	CH	CS(2)	2			1																	
55	Chileshe et al. (2018)	AU	Q(260/26)	1		1							2	2	2	1					1			
56	Sea-Lim et al. (2018)	TH	SD			1																		
57	Mahpour and Mortaheb (2018)	IR	CS(1); Q(81/81)												1									
58	Rose and Stegemann (2018)	GB	CD; CS(6); DO; SSI(21)									1	1											
59	Dunant et al. (2018)	GB	I(30)	2																	2			
60	Zaman et al. (2018)	NZ	CS(1)				1																	
61	Dunant et al. (2019)	GB	EM				1																	
62	Nußholz et al. (2019)	DK	CS(3); Q(3); SSI(3)	1		1	1	1				1		1		1	1				1			
63	Brambilla et al. (2019)	GB	CS(1)				1																	
64	Eberhardt et al. (2019)	DK	CS(1)				1																	
		Total numbers:	27	4	15	21	10	3	4	5	11	20	3	5	6	3	1	1	3	19	12	5	5

a **Country:** According to ISO 3166.

b **Research Method:** ACT: Action Research (*n* = number of case(s), if provided); AR: Archival research (*n* = number of case(s), if provided); CA: Comparative analysis; CD: Company documentation; CS: Case study (*n* = number of case(s)); DO: Direct observation (*n* = number of case(s)); DR: Document review; EM: Economic models; EX: Experiment; FGI: Focused-group interview (*n* = number of interviewee(s)); FS: Field study; GI: Group Interview; GM: Group meetings (*n* = number of attendant(s)); I: Unspecified type Interviews (*n* = number of interviewee(s)); LR: Literature review; OBS: Observation; Q: Questionnaire (*n* = number of sent Q / m = number of completed Q); S: Survey (i.e. empirical survey, etc.); SD: System dynamics; SI: Structured interviews (*n* = number of interviewee(s)); SSI: Semi-structured interviews (*n* = number of interviewee(s)); T: Theoretical study; TF: Theoretical framework; UI: Unstructured interview.

c The numbers in the table correspond with the number of drivers grouped under each sub-category.

**Table 2.** Summary of reuse barriers.

SN	Author	Cntr.^a^	Research method^b^	Categories of reuse barriers^c^																			
				Economic			Env	Organizational				Regulatory		Social						Technical			
				A: Cost	B: Market	C: Value for money	D: Energy and GHG	F: Contracts	G: Experience	H: Infrastructure	J: Management	L: Compliance	M: Incentive	O: Awareness	P: Perception	Q: Risk	R: Sustainability	S: Trust	T: Willingness	U: Deconstruction	V: Design	W: Health and safety	X: Information
1	MacKinnon (2000)	US	DR; GI; I(4); OBS	1											1								
2	Chini and Acquaye (2001)	US	EX	1								2									5		
3	Klang et al. (2003)	US	CS(1); I(10); Q(10/10)	1											1	1	1						
4	Dantata et al. (2005)	US	CS(5); LR	4																			
5	Pun and Liu (2006)	AU	TF		3																		
6	Pun et al. (2006)	AU	CS(1)	4	3							1		1								1	
7	Shaurette (2006)	US	Q(296/83)	3	2				1	2		1			1	1							
8	Guy (2006)	US	CS(4)	4								2									5	1	
9	Gorgolewski et al. (2008)	CA	AR; CS(3)	8	2						1										5		1
10	Gorgolewski (2008)	CA	AR; CS(2)	6	2				1	1		2		1	3	1			2	1	5		1
11	da Rocha and Sattler (2009)	BR	CD; CS(1); DO(5); GM(4); SSI(27)	2	1							2			1	1					1		
12	Nordby et al. (2009)	NO	CS(1)	2								1					1			2	1		
13	Jaillon and Poon (2010)	HK	AR; CS(7); DO(7); I(35); Q(84)																	1			
14	Rogers, (2011)	AE	CS(1)		1																		
15	Forsythe (2011)	AU	CS(9); DO; UI	3		1															1	2	
16	Arif et al. (2012)	IN	CS(2); SSI(15)								2		1										
17	Coelho et al. (2012)	PT	CS(15)						1														
18	Elias Özkan (2012)	TR	AR; CS; DO(21); I							2		1								1			
19	Hglmeier et al. (2013)	DE	CS(1)									1											
20	Gangolells et al. (2014)	ES	Q(658/74)						1														
21	Sansom and Avery (2014)	GB	Q(160/32)	2																		1	
22	Jaillon and Poon (2014)	HK	CS(2); LR																	2			
23	Pongiglione and Calderini (2014)	IT	AR; CS(1)	1																1	3		
24	Durão et al. (2014)	PT	CS(2)												1				1		2		
25	Chileshe et al. (2015)	AU	LR; Q(539/49); S	4					1		1	2		1	3	1			2	1		1	
26	Ferreira et al. (2015)	PT	CS(1); LR																		2		
27	Huuhka and Hakanen (2015)	FI	Q(11/11)	3	2		1					5		1	1	1			1	1	3	1	2
28	Huuhka et al. (2015)	FI	AR(276); LR									1								1	2		
29	Yeung et al. (2015)	CA	DO(4)	6		1			1		1		1								5	1	2
30	Ajayi et al. (2015)	GB	FGI(25); LR													1				1			
31	Cooper et al. (2016)	GB	CS(2); LR; SSI(17)	5																			
32	Rameezdeen et al. (2016)	AU	SSI(8)	9	2							5		2	1	2			4			2	
33	Chileshe et al. (2016b)	AU	LR; Q(539/49); SSI(6)		2				2	1		3	2	3					1	1			
34	Chinda and Ammarapala (2016)	TH	CS(2); I(6); LR	4	1					2													
35	Chileshe et al. (2016a)	AU	LR; SSI(8)	4	1	1						2	1		1	1						1	
36	Tatiya et al. (2017)	US	CS(1); LR; SI(3)	5	1															1	2	1	
37	Dunant et al. (2017)	GB	I(30); Q(24)	5	2				1	1		6			2	1		1		1			
38	Tingley et al. (2017)	GB	LR; SSI(13)	9	3	1		1				6	2	1	2	1			2	3	1	3	1
39	Yeung et al. (2017)	CA	CS(1)	2																1			
40	(Machado et al. (2018)	BR	LR							1										1	3		
41	Gálvez-Martos et al. (2018)	EU	CA		2																		
42	Huang et al. (2018)	CN	CD; LR; SSI(40)	1	1							2											
43	Brütting et al. (2019)	CH	CS(2)		1																3		
44	Sea-Lim et al. (2018)	TH	SD	2						1													
45	Rose and Stegemann (2018)	GB	CD; CS(6); DO; SSI(21)	3	4	1				1	2	1	1		1				1	1			1
46	Dunant et al. (2018)	GB	I(30)	9	1	1			1	1	1					2		1					
47	Mahpour (2018)	IR	LR; Q(6/6)												1								
48	Zaman et al. (2018)	NZ	CS(1)	1		1			1	1		1											
49	Nußholz et al. (2019)	DK	CS(3); Q(3); SSI(3)	1	3		1			1	1	2	1						1				
50	Brambilla et al. (2019)	GB	CS(1)				2													1			
51	Basta et al. (2020)	EG	CS(1); TF																	2	1		
			Total number:	115	40	7	4	1	11	15	9	49	9	10	20	14	2	2	15	24	50	15	8

a **Country:** According to ISO 3166.

b **Research Method:** ACT: Action Research (*n* = number of case(s), if provided); AR: Archival research (*n* = number of case(s), if provided); CA: Comparative analysis; CD: Company documentation; CS: Case study (*n* = number of case(s)); DO: Direct observation (*n* = number of case(s)); DR: Document review; EM: Economic models; EX: Experiment; FGI: Focused-group interview (*n* = number of interviewee(s)); FS: Field study; GI: Group Interview; GM: Group meetings (*n* = number of attendant(s)); I: Unspecified type Interviews (*n* = number of interviewee(s)); LR: Literature review; OBS: Observation; Q: Questionnaire (*n* = number of sent Q / m = number of completed Q); S: Survey (i.e. empirical survey, etc.); SD: System dynamics; SI: Structured interviews (*n* = number of interviewee(s)); SSI: Semi-structured interviews (n = number of interviewee(s)); T: Theoretical study; TF: Theoretical framework; UI: Unstructured interview.

c The numbers in the table correspond with the number of barriers grouped under each sub-category.

Throughout this study, the authors identified 57 drivers and 130 barriers affecting the reuse of building components. From a sustainability perspective, the reuse of building components has social, environmental and economic advantages (Jaillon and Poon, 2014); hence, certain factors can be categorized under these three groups. However, the successful implementation of any intervention (here, the reuse of building components) to promote sustainability in the building sector highly depends on the technical feasibility (such as durability), the regulatory enforcement (minimum performance requirements set by regulations) and the competency and willingness of the organizations engaged (knowledge, skills, infrastructure, innovation, etc.) (Nußholz et al., 2019). Therefore, an interdisciplinary approach towards sustainability becomes crucial while addressing the shortcomings in the body of knowledge on reuse (Kajikawa et al., 2014). On this basis and following Pomponi and Moncaster (2017) and Tingley et al. (2017), the authors grouped the identified reuse drivers and barriers under economic, environmental, social, technical, regulatory and organizational categories (Tables 1 and 2).

Besides, to better present the identified reuse drivers and barriers and to avoid congested tables, under each major category, the authors grouped the factors into further sub-categories, as shown in Tables 1 and 2. These sub-categories are defined based on the common characteristics of groups of factors. For instance, ‘Lower cost of reused components’ and ‘Increased cost of landfilling’ are economic drivers and are grouped under the sub-category ‘Cost’ in Table 1. It is because, in the case of the former, the lower cost of the component can decrease the total cost of the project and, in the case of the latter, landfilling is expensive and reusing the element can reduce additional costs. This approach has been pursued in the case of barriers to BCR as well.

### Reuse drivers

**Figure 5.**
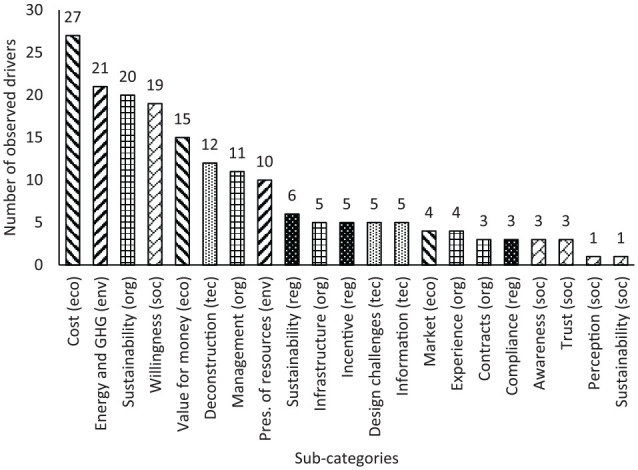
Distribution of the observed reuse drivers. eco: economic; env: environmental; org: organizational; reg: regulatory; soc: social; tec: technical.

Figure 5 shows the distribution of the observed drivers in the reviewed papers. According to this figure, the principal identified drivers are economic (25%), organizational (23%), environmental (17%) and social (15%). The sub-categories of the factors shown in this figure present a similar trend between main categories and sub-categories. Among the drivers, ‘cost’ is the most reported sub-category, followed by ‘energy and GHG’, ‘organizational sustainability’ and ‘willingness’ sub-category of drivers. These observations are discussed further in the following subsections.

#### Economic drivers

From the reviewed articles, it is observed that the potential cost savings as the result of using recovered building components can promote reuse. For example, according to Chileshe et al., (2018), da Rocha and Sattler (2009), Dunant et al. (2017), Gorgolewski et al. (2008), Klang et al. (2003) and MacKinnon (2000), the lower price of the reused components can contribute to the cost savings in the construction projects. Likewise, according to Cooper et al. (2016), reusing steel sections results in the purchase of fewer new steel sections. If the price for the reused components is attractive, the demand for them can increase (Klang et al., 2003), which in the long run supports the growth of a reuse market (Chileshe et al., 2018; da Rocha and Sattler, 2009; Tingley et al., 2017) and increases the revenue from the resale of these components (Dantata et al., 2005; da Rocha and Sattler, 2009; Dunant et al., 2017; Klang et al., 2003; Sea-Lim et al., 2018). Moreover, the increased cost of landfilling can act as a reuse driver because it increases the disposal cost of CDW (Dantata et al., 2005; Gorgolewski, 2008; Chileshe et al., 2016a; Chinda and Ammarapala, 2016). By reusing the recovered building components, this extra cost can be decreased (Pun et al., 2006). However, these factors highly depend on the geographic location of the building, which might have an opposing effect on reuse. For instance, (Huang et al., 2018) report that the lower cost of landfilling is an impediment to reuse. The study is performed in China, where cheap landfilling discourages choosing other waste treatment options such as reuse or recycling.

#### Organizational drivers

According to the literature, reducing CDW generated by the firms (Aye et al., 2012; Densley Tingley et al., 2012; Guy, 2006; Pun et al., 2006; Schultmann and Sunke, 2007 (among others^1^)) and promoting the green image of the companies to improve competitiveness (Chileshe et al., 2016a, 2016b; Chinda and Ammarapala, 2016; Durão et al., 2014; Rogers, 2011 (among others)) rank the highest among all other organizational drivers.

One method to increase the reuse rates by the organizations is through integrating reuse in the design process of new projects (Gorgolewski, 2008; Gorgolewski et al., 2008; Rogers, 2011; Tingley et al., 2017 (among others)). As a result, and to support this idea, some articles suggest that by integrating reuse in the contractual requirements, reuse rates will increase (Gorgolewski, 2008; Gorgolewski et al., 2008; MacKinnon, 2000). Also, a reclaimed components management coordinator (Gorgolewski, 2008; Tingley et al., 2017) and the knowledge of a known list of structural components to reuse early on in the design phase are recommended in order to potentially increase the adoption of reuse by the firms (Gorgolewski, 2008; Rose and Stegemann, 2018). The latter can be facilitated by coordination between the owners of the demolition site and the new building. However, in many instances, this coordination never happens (Dunant et al., 2018, Nußholz et al., 2019). One solution, as observed by Nußholz et al. (2019), is using companies’ entrepreneurial activities to integrate circular principles. According to this study, a Danish company involved in brick reuse could overcome certain limitations by changing its business model by integrating deconstruction into its scope to safeguard a more sustainable supply of the reused bricks.

Training operators for effective deconstruction (Dantata et al., 2005; Elias Özkan, 2012; Shaurette, 2006), availability of space for the storage of the reusable components after deconstruction (Rogers, 2011) and knowledge and experience in using reused components (Tingley et al., 2017), as well as proper separation of the reusable components after deconstruction (Ajayi et al., 2017; Ding et al., 2016; Elias Özkan, 2012; Rogers, 2011), are among other factors driving reuse.

#### Social drivers

Factors such as society’s environmental concerns (Chileshe et al., 2016a) or the increased awareness of the full benefits of reuse among the stakeholders (MacKinnon, 2000) are identified as drivers to reuse. Nußholz et al. (2019) reports recognition of reuse in the public debate can enhance public awareness and promotes reuse.

However, from a social perspective, positive perception and willingness of the stakeholders such as clients (Arif et al., 2012; Dunant et al., 2017, 2018; Gorgolewski, 2008; Gorgolewski et al., 2008; Sansom and Avery, 2014; Shaurette, 2006; ), designers (Dunant et al., 2017, 2018; Gorgolewski, 2008; Gorgolewski et al., 2008; Rameezdeen et al., 2016; Tingley et al., 2017) and contractors (Chileshe et al., 2016b, 2018; Dunant et al., 2017; Gorgolewski et al., 2008; Rogers, 2011) to integrate reused components into their projects are determining.

Unlike new building components that can be sourced from the market with proper quality certificates, salvaged building components are usually not available off the shelf and cannot be trusted. However, according to a few articles, informality and good relationships among the stakeholders are reported to help overcome this challenge and promote reuse (Chileshe et al., 2016b; da Rocha and Sattler, 2009; Shaurette, 2006).

#### Environmental drivers

One potential reuse driver is the scarcity of landfilling sites, which helps the environment by avoiding the dumping of reusable waste into landfills (Chau et al., 2012; Chinda and Ammarapala, 2016). According to the literature, reuse can decrease the use of virgin materials and water consumption (Aye et al., 2012; Densley Tingley et al., 2012; Sára et al., 2001; Tingley et al., 2017; Yeung et al., 2017). As mentioned in the introduction, because of the considerable advantages of reuse, components reuse can improve the environmental footprint of buildings worldwide. By reusing building components, embodied energy and carbon of construction can be decreased (Brütting et al., 2019; Klang et al., 2003; Tingley et al., 2017; Yeung et al., 2017 (among others)). Brütting et al. (2019) show that a structure made with reused steel sections have considerably lower embodied energy and CO_2_ emissions. In their study, the authors developed a discrete structural optimization method to reuse the existing stock of the steel sections. They used Life Cycle Analysis (LCA) to compare the environmental impacts of conventional design with the proposed method (Brütting et al., 2019).

#### Other drivers

Based on the reviewed articles, deconstruction instead of demolition can enhance the reusability of the recovered components (Gorgolewski et al., 2008; Hglmeier et al., 2013; Pongiglione and Calderini, 2014; Yeung et al., 2015 (among others)). According to Gorgolewski (2008), Gorgolewski et al. (2008) and Pongiglione and Calderini (2014), the availability of information about the characteristics, details, certificates and drawings of the recovered building components can positively contribute to increasing the reuse rates as well.

In projects with recovered building components, the proper estimation of the required sizes and lengths at the beginning of the design phase is reported to promote reuse (Gorgolewski et al., 2008). Some articles advise that reusing the recovered components, such as the structural components, to serve the same purpose (for instance, similar loads) has a positive impact on the success of this intervention (Gorgolewski, 2008; Gorgolewski et al., 2008; Pongiglione and Calderini, 2014).

The environmental policies (Chileshe et al., 2018) and green building rating systems such as Building Research Establishment Environmental Assessment Method (BREEAM) and Leadership in Energy and Environmental Design (LEED) are reported to have a positive impact on reuse rates (Shaurette, 2006; Gorgolewski, 2008). The availability of regulatory and financial incentives to encourage deconstruction and reuse, as well as the existence of regulations supporting these interventions, can potentially promote reuse (Chileshe et al., 2018). However, according to the reviewed articles, such ordinances are currently not available (Chileshe et al., 2016b, 2016a; Rose and Stegemann, 2018; Tingley et al., 2017; Yeung et al., 2015).

### Reuse barriers

**Figure 6.**
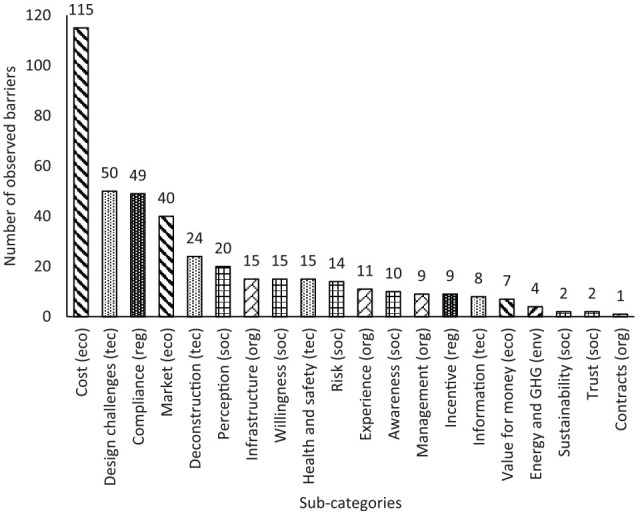
Distribution of the observed reuse barriers. eco: economic; env: environmental; org: organizational; reg: regulatory; soc: social; tec: technical.

Figure 6 shows the distribution of the observed barriers in the reviewed papers. According to this figure, the identified barriers are primarily economic barriers (39%), followed by the technical (23%) and social barriers (15%). The sub-category of the factors shown in this figure reveals additional information about the observations. Among the identified factors, ‘cost’ is the most reported sub-category of barriers, followed by ‘design challenges’, ‘compliance’, ‘market’, ‘deconstruction’, and ‘perception’. However, unlike the main categories, the third rank in the sub-categories, ‘compliance’, is a regulatory barrier. These observations are discussed further in the following sections.

#### Economic barriers

Although deconstruction can increase the reusability of the recovered building components (Addis, 2006; Munroe et al., 2006), it is believed to be more labour intensive (Chileshe et al., 2015; Gorgolewski et al., 2008; Rameezdeen et al., 2016). Dantata et al. (2005) highlight that the time required to deconstruct a 90 to 180 m2 building is three to five times higher than the time needed for the demolition of the same building. According to the reviewed articles, the time required for deconstruction and reuse, and the consequent project scheduling, is one of the main barriers to reuse (Dantata et al., 2005; Gorgolewski, 2008; Gorgolewski et al., 2008; MacKinnon, 2000; Shaurette, 2006 (among others)). It is because there is usually a high pressure to complete construction projects as early as possible (Chinda and Ammarapala, 2016). The tight project schedule negatively affects the efficient disassembly of the existing buildings and lowers the chance for the recovery of reusable building components (Sansom and Avery, 2014).

During the deconstruction phase, more time is required to carefully remove and sort the recovered building components (Gorgolewski, 2008), which increases the cost of sorting (Rameezdeen et al., 2016). Sometimes the deconstruction time extends beyond anticipations because of issues such as a lack of space for equipment, complexity of the building design and the geographic location of the building (Tatiya et al., 2017). These extra charges can yield a higher deconstruction cost (when compared with the demolition of the same building) (Chileshe et al., 2015; Dantata et al., 2005; Dunant et al., 2018; Rose and Stegemann, 2018; Tingley et al., 2017; Yeung et al., 2015) and eventually increase the price of the recovered components (Chileshe et al., 2015; Chileshe et al., 2016a; Dunant et al., 2018; Rameezdeen et al., 2016; Shaurette, 2006; Tingley et al., 2017).

Another economic barrier to the BCR is the higher cost of design with the reused components (Dunant et al., 2017; Gorgolewski, 2008; Gorgolewski et al., 2008). It is because the design team needs to put in extra efforts to find the reused elements (Gorgolewski et al., 2008), and the design needs to remain as flexible as possible (Gorgolewski et al., 2008). Sometimes it is required to purchase the identified reused components early in the project (Gorgolewski, 2008; Gorgolewski et al., 2008) to cope with uncertainty about the timely availability of the desired elements (Chileshe et al., 2015; Gorgolewski et al., 2008). Consequently, this practise may raise cash flow problems and increase the overall cost of the project due to additional storage costs, which is another barrier to the BCR (Chinda and Ammarapala, 2016; da Rocha and Sattler, 2009; Gorgolewski, 2008; Gorgolewski et al., 2008; Yeung et al., 2015 (among others)).

All the above explain the increased labour cost (Chinda and Ammarapala, 2016; Dantata et al., 2005; Gorgolewski et al., 2008; Klang et al., 2003; Rameezdeen et al., 2016; Shaurette, 2006 (among others)), transportation cost (da Rocha and Sattler, 2009; Gorgolewski, 2008; Gorgolewski et al., 2008; Pongiglione and Calderini, 2014; Rameezdeen et al., 2016; Yeung et al., 2015 (among others)) and storage cost associated with deconstruction and reuse, which are identified as barriers to the BCR in several articles.

In some cases, the fabrication cost of the recovered building components might be higher than the fabrication cost of the new elements (Dunant et al., 2017, 2018; Tingley et al., 2017). Dunant et al. (2017) explain that because reused steel components are associated with existing connections, holes, stiffeners, welds, end-plates, etc., the preparation of these components might increase the overall cost of fabrication because of the extra time, labour and machinery required. Other additional charges which can increase the overall price of the recovered components are cost of testing (Dunant et al., 2018; Gorgolewski, 2008; Rameezdeen et al., 2016; Tingley et al., 2017; Yeung et al., 2015), cost of treatment of the salvaged parts (Chini and Acquaye, 2001; Dunant et al., 2018; Huuhka and Hakanen, 2015), cost of insurance (Tingley et al., 2017) and cost of marketing for the recovered building components (Dantata et al., 2005).

Another barrier to reuse, as reported in several articles, is the lack of an established market for the reused building components (Chileshe et al., 2016a, 2016b; Chinda and Ammarapala, 2016; Gorgolewski, 2008; Gorgolewski et al., 2008; Rameezdeen et al., 2016; Shaurette, 2006 (among others)). This factor, which is partially the outcome of the tight project schedules (Tatiya et al., 2017), results in the lack of sufficient supply of reused components with the desired characteristics (dimension, quality, etc.) (Brütting et al., 2019; da Rocha and Sattler, 2009; Dunant et al., 2017; Gorgolewski, 2008; Rose and Stegemann, 2018; Tingley et al., 2017). According to Dunant et al. (2018), the above restriction encourages the contractors to sell their reusable waste to the recycling companies regardless of their high quality (Huuhka and Hakanen, 2015; Sansom and Avery, 2014; Tingley et al., 2017; Yeung et al., 2015, 2017). If the demand for the reused building components increases (Chileshe et al., 2016b), the market for these products can grow sustainably. In contrast, lack of demand (Chileshe et al., 2016b; Huuhka and Hakanen, 2015; Rogers, 2011; Shaurette, 2006; Tingley et al., 2017) or uncertainty about the need for the reused components (Rose and Stegemann, 2018) causes scepticism about the revenue from the reused components resale (Chileshe et al., 2016a; Dunant et al., 2018; Rose and Stegemann, 2018; Yeung et al., 2015). All the above negatively affect the chance for the growth of a reuse market. With an underdeveloped reuse market, the supply chain remains fragmented and information about the supply and demand cannot be shared, which further decreases the reuse rates (Gorgolewski et al., 2008; Rameezdeen et al., 2016; Rose and Stegemann, 2018).

According to the literature, higher deconstruction costs can hinder its application (Chileshe et al., 2015; Dantata et al., 2005, 2018, Rose and Stegemann, 2018, Yeung et al., 2015, Tatiya et al., 2017; Tingley et al., 2017) and might elevate the financial risks associated with deconstruction and reuse (Rameezdeen et al., 2016). However, this finding is in contrast with the observations in da Rocha and Sattler (2009). According to this study, in Brazil, the cost of deconstruction is lower than demolition due to the low cost of manual labour and the high demand for demolition products (da Rocha and Sattler, 2009). In a separate study, Dantata et al. (2005) suggest that if the productivity of the deconstruction team increases or the wages decrease or the disposal cost rises, the overall cost of deconstruction decreases, and it becomes a desirable option (in Massachusetts). Therefore, it can be concluded that the socio-economic context of the location of a building can convert some barriers to drivers and vice-versa.

#### Technical barriers

Ajayi et al. (2015) suggest that by integrating DfD during the design stage of a building, the recovery of building components for reuse would be facilitated. According to the literature, the lack of such an intervention is a barrier to reuse (Ajayi et al., 2015; Chileshe et al., 2015, 2016b; Dunant et al., 2017; Huuhka and Hakanen, 2015; Tatiya et al., 2017 (among others)). Some outcomes of this design gap are permanent joints (welding, etc.) (Gorgolewski, 2008; Pongiglione and Calderini, 2014; Tingley et al., 2017), composite joints (Tingley et al., 2017) and hard to access connections (Tingley et al., 2017), which can negatively affect deconstruction and make the recovery of the building components challenging (Huuhka et al., 2015).

Because deconstruction is not considered at the design stage, building components are prone to more damage during the deconstruction phase (Chini and Acquaye, 2001; Gorgolewski, 2008; Pongiglione and Calderini, 2014). Damages to the reused building components can decrease the quality of the elements and affect their reusability (da Rocha and Sattler, 2009; Durão et al., 2014; Huuhka and Hakanen, 2015; Tatiya et al., 2017). Damages can also happen as the result of corrosion (Chini and Acquaye, 2001; Huuhka et al., 2015; Yeung et al., 2015), post-production modiﬁcations (holes for ductwork, etc.) (Chini and Acquaye, 2001; Yeung et al., 2015), presence of water (Yeung et al., 2015; Tatiya et al., 2017), exposure to weather conditions (Huuhka and Hakanen, 2015), fire (Yeung et al., 2015; Tatiya et al., 2017), refurbishment (nail removal, etc.) (Chini and Acquaye, 2001), by living organisms (termites, bacterial attack, etc.) (Chini and Acquaye, 2001), fatigue (Yeung et al., 2015), frost (Huuhka et al., 2015), degradation (Durão et al., 2014), type of joints (Gorgolewski, 2008) and during the storage and transportation of recovered components (Gorgolewski, 2008; Yeung et al., 2015, etc.).

Difficulty in designing with the reused components is another barrier to the widespread reuse of the building components (Brütting et al., 2019; Gorgolewski et al., 2008; Pongiglione and Calderini, 2014; Tingley et al., 2017). As discussed earlier, the design of the new buildings with reused building components needs to remain flexible. This is because the design should be able to accommodate alternative dimensions of the reused components due to the uncertainty in the availability of the desired sections (Gorgolewski, 2008; Gorgolewski et al., 2008). Brütting et al. (2019) argue that unlike structures made out of new steel sections, where components with different cross-sections and lengths can be fabricated to the required shape, in the case of the reused steel sections, this luxury doesn’t exist and the properties of the available components dictate the structure’s geometry.

Pongiglione and Calderini (2014) discuss that in the process of designing a new structure using the recovered components, due to architectural and structural reasons, new structural elements should be used as well. However, to secure the safety of such structures, the new components should be over-dimensioned, which eventually results in overdesigned structures (Brütting et al., 2019; Gorgolewski, 2008; Gorgolewski et al., 2008; Pongiglione and Calderini, 2014). This is either because of the lower strength of the reused components or that the remaining capacity of the reused components is unknown (Huuhka and Hakanen, 2015; Yeung et al., 2015). The latter happens when the information about the characteristics, details, certificates and drawings of the reused components are not available (Gorgolewski, 2008; Gorgolewski et al., 2008; Huuhka and Hakanen, 2015; Rose and Stegemann, 2018; Tingley et al., 2017; Yeung et al., 2015). Other design challenges while reusing recovered building components are designing with long spans (because such elements might not be readily available) (Brütting et al., 2019; Gorgolewski et al., 2008; Huuhka and Hakanen, 2015), difference in the loading requirements of the old and the new buildings (Gorgolewski et al., 2008), and the mismatch between the old spans and the new features (Huuhka and Hakanen, 2015).

Additional health and safety precautions necessary for deconstruction, component recovery, and reuse are some other technical barriers to reuse (Chileshe et al., 2015, 2016a; Huuhka and Hakanen, 2015; Rameezdeen et al., 2016; Sansom and Avery, 2014; Tingley et al., 2017; Yeung et al., 2015). It is because, during the deconstruction of a building, or while treating a component for reuse, there is a risk of encountering hazardous, banned or contaminating coatings on the reused components (Rameezdeen et al., 2016; Tatiya et al., 2017; Tingley et al., 2017). In the case of facing hazardous materials such as lead or asbestos, speciﬁc procedures and licensed contractors are required (Rameezdeen et al., 2016).

#### Social barriers

The negative perception of the stakeholders about the reused building components can act as a barrier to reuse (Chileshe et al., 2015, 2016a; Huuhka and Hakanen, 2015, Klang et al., 2003, MacKinnon, 2000, Rameezdeen et al., 2016 (among others)). One reason behind this is the visual appearance of the reused components that might be interpreted as lower quality when compared with a new element (Dunant et al., 2017; Durão et al., 2014; Tingley et al., 2017). For instance, Durão et al. (2014) report that the architects refuse to use recovered wood in visible places due to its poor appearance. However, the visual appearance can be a point of further discussion since it is highly subjective and can be attractive to some people (Nußholz et al., 2019). Another reason for this negative perception, and, at a larger scale, the construction sector’s resistance against reuse (Durão et al., 2014; Gorgolewski, 2008; Rameezdeen et al., 2016; Tingley et al., 2017), stems from the potential risks perceived by the stakeholders during deconstruction or while using the recovered building components (Chileshe et al., 2015; Dunant et al., 2017; Gorgolewski, 2008; Rameezdeen et al., 2016; Shaurette, 2006; Tingley et al., 2017).

The occupational health concerns (Klang et al., 2003, Rameezdeen et al., 2016), liability and fear (da Rocha and Sattler, 2009), lack of trust to the supplier of the reused components (Dunant et al., 2017, 2018) and unsatisfactory working environment during the treatment of the reused components (Klang et al., 2003) can all worsen the lack of interest to integrate the reused components in the projects (Chileshe et al., 2016b, Rameezdeen et al., 2016). Among the stakeholders, the perceptions of clients (Chileshe et al., 2015; da Rocha and Sattler, 2009; Dunant et al., 2017; Rose and Stegemann, 2018), contractors (Gorgolewski, 2008; Shaurette, 2006) and designers (Gorgolewski, 2008) have a higher impact on the successful integration of recovered components into a new building. However, if the client does not support reuse (Huuhka and Hakanen, 2015; Rameezdeen et al., 2016; Rose and Stegemann, 2018; Tingley et al., 2017), there is a much smaller chance that the designers or contractors will risk the project by introducing such components. On the other hand, according to Gorgolewski (2008), if the client is motivated to use the reused building components, the barriers such as the unwillingness of the design team (Chileshe et al., 2015; Rameezdeen et al., 2016) or the contractors (Gorgolewski, 2008) can be handled effectively. Nevertheless, the inequality in the distribution of risk among the stakeholders (Dunant et al., 2018) can still challenge motivated clients and architects.

Gorgolewski (2008) argues that while choosing deconstruction to remove the existing buildings improves the supply of the reused components, due to the perceived economic and programming reasons, it is not yet a preferred option among the contractors (Gorgolewski, 2008). One reason for such reluctance is because the stakeholders are unaware of the full benefits of deconstruction and reuse (Chileshe et al., 2015, 2016b; Gorgolewski, 2008; Huuhka and Hakanen, 2015; Rameezdeen et al., 2016). As mentioned earlier, some of the benefits of deconstruction and reuse are the cost savings and reduced environmental pollution. Therefore, educating the stakeholders on the advantages of deconstruction and reuse, as identified by Chileshe et al. (2015) and Gorgolewski (2008), could be an effective measure to cope with some of the social resistance against reuse.

#### Regulatory barriers

One of the challenges ahead of reuse is that the existing regulations do not support deconstruction and reuse (Chileshe et al., 2015, 2016b; Gorgolewski, 2008, Hglmeier et al., 2013; Huuhka and Hakanen, 2015; Huuhka et al., 2015; Rameezdeen et al., 2016 (among others)). Rameezdeen et al. (2016) argue that bureaucracy is a barrier ahead of necessary approvals for deconstruction projects in South Australia. According to this study, even after getting approvals for deconstruction, since existing regulations do not allow the storage of the salvaged components and consider them as waste (Rameezdeen et al., 2016), the reuse of the recovered components is hindered. This study suggests that governments should support the reuse of recovered components in the new constructions (Rameezdeen et al., 2016); however, in reality, this is not the case (Chileshe et al., 2016b, 2016a). Rameezdeen et al. (2016) further discuss that, while regulations support recycled-content products, due to the inconsistency and lack of coordination among the regulatory bodies (Chileshe et al., 2016a; Rameezdeen et al., 2016), regulatory agencies have a prohibitive approach towards deconstruction and reuse. It should be noted that these studies focus on the Australian construction sector, and the results should be considered cautiously (Chileshe et al., 2016a, 2016b, Rameezdeen et al., 2016).

Lack of quality certificates for the reused components can negatively affect reuse (Chini and Acquaye, 2001). Dunant et al. (2017) explore this barrier by highlighting the need for the traceability of the steel sections, which is essential to certify, fabricate and erect the segments. Usually, the traceability of the reused steel sections cannot be guaranteed (Dunant et al., 2017; Tingley et al., 2017), and in many instances, all the segments need to be tested to certify their properties and assure their quality. However, according to this study, in the case of stricter requirements on Conformité Européene (CE) marking (Dunant et al., 2017; Tingley et al., 2017), even the individual testing fails to certify the reused components.

Lack of confidence in the quality of the reused components negatively affects reuse in new constructions (Ajayi et al., 2015; Chileshe et al., 2015, 2016b, 2016a; Shaurette, 2006 (among others)). Huang et al. (2018) observed that there is a negative attitude towards using recovered construction and demolition waste among the building construction companies because of the lack of guarantees for these components. According to the reviewed articles, currently, there are no standards to certify the quality of the reused components (Chini and Acquaye, 2001, Dunant et al., 2017, Huang et al., 2018). Therefore, the lack of procedures to evaluate and guarantee the performance of reused components (Shaurette, 2006, Tingley et al., 2017), and the fact that the existing codes, standards and procedures do not consider BCR (Gorgolewski, 2008; Huuhka and Hakanen, 2015; Rameezdeen et al., 2016; Tingley et al., 2017), further decrease the reuse rate in construction.

#### Organizational barriers

Because deconstruction and reuse are still uncommon practises (Dunant et al., 2017, 2018), the number of companies with experience in deconstruction and reuse is low (Chileshe et al., 2016b). According to the literature, the lack of skills, experience, and knowledge in deconstruction, salvage and using reused components negatively affect the reuse of the building components (Chileshe et al., 2015, 2016b; Gorgolewski, 2008, Shaurette, 2006, Yeung et al., 2015). Unlike demolition, deconstruction requires enough space for the storage, sorting and treatment of the recovered building components. However, an inexperienced contractor cannot correctly estimate the space required for the storage of the recovered components after deconstruction. This lack of space for storage (Chinda and Ammarapala, 2016; Dunant et al., 2017, 2018; Gorgolewski, 2008; Rose and Stegemann, 2018; Shaurette, 2006) results in the transportation and storage of the recovered components at a different location and would increase the overall cost of the reused elements.

Lack of systems thinking (Rose and Stegemann, 2018), ownership (Arif et al., 2012) and the integration of reuse in the design process of the new projects (Rose and Stegemann, 2018) are factors identified that decrease the reuse rates in the building sector. Yeung et al. (2015) highlight the importance of a decision-making framework in informing the contractors and clients regarding when alternative reuse options should be investigated. According to this study, this decision-making framework helps making informed decisions about deconstruction and reuse and maximizes the advantages of potential reuse by identifying the necessary steps to be taken by the stakeholders (Yeung et al., 2015). Other observed organizational barriers are proprietary lock-ins (Tingley et al., 2017), the need for infrastructure and equipment to perform deconstruction (Chileshe et al., 2016b, Sea-Lim et al., 2018; Shaurette, 2006) and inconsistency in waste management practises (Arif et al., 2012).

#### Environmental barriers

Although component reuse is identified as a sustainable end-of-life treatment of the superstructure of a building (Brütting et al., 2019; Klang et al., 2003; Tingley et al., 2017; Yeung et al., 2017), there are concerns regarding the adverse effects of this practise due to the increased GHG emissions related to deconstruction activities and the transportation of recovered elements (Brambilla et al., 2019; Huuhka and Hakanen, 2015; Nußholz et al., 2019).

Brambilla et al. (2019) performed a study to evaluate the environmental impacts of various steel-concrete composite floor systems. In this study, the authors performed a comparative LCA and compared the four composite connections, including a novel a demountable steel-concrete composite ﬂoor system and three conventional systems. The authors concluded that a transport distance between 20 km and 200 km has no significant impact on environmental advantages achieved by the demountable system. However, they concluded that a distance of 1000 km could diminish the environmental benefits achieved by this system. The authors also discussed that the deconstruction of the demountable composite structure takes more time compared with demolition, which results in the emission of higher amounts of GHGs since the heavy machinery and equipment need to operate for longer periods (Brambilla et al., 2019).

### Prioritizing reuse barriers

Previous observations provide an insight into the challenges ahead of component reuse in the building sector; however, prioritizing them needs a further investigation about the inter-dependency of these factors. Reviewing the co-occurrences of data is a way to identify the impact of a variety of variables in a research topic on one another and to reveal their potential correlations. Moreover, identifying the correlation between the key variables helps in better devising solutions to achieve the objectives of the study (Rameezdeen et al., 2016; van Eck and Waltman, 2009). In this section, we analyse the inter-relationship between the observed barriers through developing the co-occurrence of all the 20 sub-categories available in Table 2.

In this study, we considered a binary approach for the presence (1) or the absence (0) of the sub-category of the barriers in Table 2 to identify their co-occurrences and eventually develop their correlations. It means that if in Table 2, under a particular sub-category for a specific paper, no barrier is observed, value 0, which means absence, is considered. On the other hand, the available observations (regardless of their number) are converted to 1.

Table 3 shows the co-occurrence of the sub-categories of reuse barriers in the reviewed articles. For example, sub-category A and sub-category B (AB) appear 16 times together in all the articles reviewed in this study. To analyse the correlation between the sub-categories, we also developed the co-occurrence index (C-Index) of the pairs of the sub-categories. In this work, we calculated the c-Index using the software ‘R’ (version 3.6.1) (R Core Team, 2019) through the ‘jaccard’ package (Chung et al., 2018), which is based on equation 1 (Atlas.ti, 2014). In this equation, n12 is the co-occurrence frequency of the two sub-categories (the number of times the two sub-categories show-up together; hence it is not equal to n1+n2), and n1 and n2 are the total numbers of occurrences of each of the sub-categories in all the studies. C-Index varies from 0 to 1, with 1 showing the highest correlation and 0 indicating no relationship. The null hypothesis is that there is no correlation between the pairs of the sub-categories. To test the null hypothesis, we use the *p*-value through the embedded test in the ‘jaccard’ package (jaccard.test.exact) (Chung et al., 2018). If the *p*-value is less than 0.05, then the null hypothesis is false and, statistically, there is a correlation between the pairs of the sub-categories (James et al., 2017).

(1) $$C-Index=\frac{n12}{\left(n1+n2\right)-n12}$$

**Table 3.** Co-occurrence of the sub-categories of reuse barriers.

Code	Economic			Env	Organizational				Regul.		Social						Technical			
	A: Cost	B: Market	C: Value for money	D: Energy and GHG	F: Contracts	G: Experience	H: Infrastructure	J: Management	L: Compliance	M: Incentive	O: Awareness	P: Perception	Q: Risk	R: Sustainability	S: Trust	T: Willingness	U: Deconstruction	V: Design challenges	W: Health and safety	X: Information
A	−	16	7	2	1	7	9	6	17	5	6	12	11	2	2	7	10	12	11	6
B		−	4	2	1	5	8	4	13	5	6	9	9	0	2	7	7	7	6	5
C			−	0	1	3	3	3	4	4	1	3	3	0	1	2	2	3	4	3
D				−	0	0	1	1	2	1	1	1	1	0	0	2	2	1	1	1
F					−	0	0	0	1	1	1	1	1	0	0	1	1	1	1	1
G						−	6	3	6	2	3	4	5	0	2	3	4	2	2	2
H							−	3	8	3	2	4	4	0	2	4	6	2	0	2
J								−	3	4	1	2	2	0	1	3	2	2	2	3
L									−	5	7	10	9	1	1	8	10	8	7	4
M										−	2	3	2	0	0	4	3	2	3	3
O											−	5	5	0	0	6	5	3	5	3
P												−	10	1	1	7	6	5	5	4
Q													−	1	2	5	6	4	5	3
R														−	0	0	1	1	0	0
S															−	0	1	0	0	0
T																−	6	4	4	4
U																	−	9	4	4
V																		−	6	5
W																			−	3

In Table 3, the highlighted cells represent the high levels of co-occurrence between the sub-categories. The corresponding c-index of these pairs of sub-categories of the barriers are sorted and listed in Table 4. Also, the *p*-value, which indicates if the correlation is significant or not (James et al., 2017), is listed against each of the pairs.

**Table 4.** C-Indices of the correlation between major sub-categories.

Seq. No	Code	Sub-category pair	C-Index	*P*-value
1	PQ	Perception and Risk	0.63	<0.00001^*^
2	AL	Cost and Compliance	0.49	0.007^*^
3	BL	Market and Compliance	0.45	0.006^*^
4	AB	Cost and Market	0.44	0.04^*^
5	LP	Compliance and Perception	0.40	0.004^*^
6	BQ	Market and Risk	0.38	0.004^*^
7	LQ	Compliance and Risk	0.38	0.004^*^
8	AP	Cost and Perception	0.36	0.02^*^
9	AW	Cost and Health and safety	0.35	0.001^*^
10	BP	Market and Perception	0.35	0.02^*^
11	AQ	Cost and Risk	0.34	0.007^*^
12	LU	Compliance and Deconstruction	0.33	0.2
13	AV	Cost and Design challenges	0.32	0.5
14	UV	Deconstruction and Design challenges	0.32	0.1
15	AH	Cost and Infrastructure	0.26	0.2
16	AU	Cost and Deconstruction	0.25	0.4

* Denotes a significant correlation (*p*<0.05).

According to Table 4, there is a significant correlation between perception and risk, with the c-index of 0.63 ranking the highest among other sub-categories. It indicates that the perception of the stakeholders about reuse is affected by the potential risks associated with this intervention. Perception co-occurs with compliance, cost and market as well (all are significant with *p*-values 0.004, 0.02 and 0.02, respectively). It reveals the importance of addressing the economic and regulatory obstacles to promote reuse among the stakeholders. The second and third highest ranks belong to the cost and compliance as well as market and compliance, with the c-indices of 0.49 and 0.45, respectively. It shows that an established reused market requires products with reasonable prices complying with state-of-the-art codes and regulations to be offered. On the other hand, the existence of ordinances, as well as the best practises on the reused components, would help the growth of a reuse market.

The fourth highest rank belongs to cost and market with a c-index of 0.44. It indicates that without a competitive price, a well-established market for reused elements is unlikely to grow. Moreover, it depicts that the growth of the reused components market can help to make the cost of reused components more competitive. However, the correlation between these two sub-categories is not very significant (*p*-value close to 0.05). It is interesting because, in most of the reviewed papers, both sub-categories are repeated. It can be further interpreted that these two sub-categories are similar, and no special consideration for prioritizing this pair is required as the improvement in one promotes the other one.

From Table 4, we can observe that the social, economic, and regulatory barriers co-occur frequently. Therefore, it seems that any further action to promote reuse should prioritize actions to be taken under these themes. Notwithstanding, this result is different from our initial observation in Figure 6, where the economic factors were ranked the highest, followed by the technical, social, regulatory and organizational barriers.

## Discussion

The observed environmental advantages of reuse indicate that this intervention is an effective strategy that should receive more attention to reduce the environmental footprint of the building sector.

From an economic perspective, the advantages of reuse in terms of cost savings and profit are key drivers. According to the reviewed articles, economic barriers can be categorized into supply chain level, component level and project level. At the supply chain level, in the absence of a mature reuse market, the sustainable supply of recovered components for use in the superstructure of a building is challenging. Although some innovative companies, such as Gamle Mursten in Denmark, integrate deconstruction into its core business (Nußholz et al., 2019), most companies are reluctant to change their business model. Hence, as advised by Dunant et al. (2018) and Nußholz et al. (2019), close cooperation between construction and demolition companies can address this barrier. At the component and project levels, a strict financial risk assessment at the beginning of the project should be performed. Because this intervention is rather new, the availability of resources to decrease the financial risks would be helpful (Gorgolewski, 2008; Tingley et al., 2017). Such financial incentives have the potential to promote deconstruction and reuse activities, could help the growth of reuse markets and potentially make the price of the recovered elements more competitive (see Table 4).

Alternatively, other attempts could be made to make the cost of the recovered components competitive. One possible solution is following the successful example of increasing the landfilling tax in the UK (Defra, 2007, 2019). Considering the waste hierarchy, if the cost of other waste treatment options increases in favour of reuse, the additional costs due to deconstruction, treatment and testing could be compensated. However, there are reports of illegal landfilling in reaction to the increased landfilling taxes (da Rocha and Sattler, 2009; Rameezdeen et al., 2016). Therefore, further research in different geographical locations should be conducted to recognize the mechanisms leading to such behaviour and provide guidelines to prevent it.

From a social perspective, the factors affecting reuse can be categorized into perception, awareness and risks. Most of the discussions in the literature from a social perspective are focused on the perception and willingness of the stakeholders regarding reuse and are less focused on the advantages of reuse for the general public. Therefore, further research should be conducted to establish the benefits of reuse for society. Nevertheless, the negative perception of the stakeholders towards reuse is recognized in the literature as an impediment to its adoption in the building sector. Based on Table 4, this negative perception is associated with the perceived risks at different stages of projects with recovered building components as well as the need for compliance with the regulatory requirements and is fueled by the concerns about the health and safety of the stakeholders. Therefore, steps should be taken to improve the perception of the stakeholders about the recovered building components. For instance, the development of standard test procedures to test, evaluate and certify the recovered building components can positively contribute to this attempt. Such standards and guidelines can address the reported concerns and resistances in the construction sector against the recovered building components and help the growth of a reuse market by offering quality products.

The regulatory barriers can be categorized into incentive level and compliance level, which the advantages of the availability of regulatory incentives were discussed earlier. At the policy level, the reported regulatory barriers highlight that the existing codes and regulations do not consider deconstruction and reuse, which, in the long run, inhibit the integration of the recovered building components in the superstructure of the buildings. Moreover, as discussed earlier, the existing standards only certify new components and not the recovered elements. According to the previous section, the capability of suppliers in offering second-hand components with proper quality certificates and guarantees could potentially help the growth of a reuse market (Table 4). In this regard, one possible solution is the development of new standards to certify recovered building components. An example of the successful development of certifying standards is provided by Nußholz et al. (2019). In this study, the case study companies developed certifying standards to assure the quality of their products. Moreover, proper standards and procedures should be developed for the effective deconstruction of the existing buildings and guide designers to integrate the recovered building components into the design of new buildings. Because of the variety of building designs in different periods and locations, proper databases for the existing buildings should be developed to assist such guidelines. These databases should contain the historical reports for each building, including the refurbishments, fire, extensions and the potential end-of-life treatment plans.

According to the literature, the advantages of reuse in reducing the CDW and increasing the competitiveness of the firms are key organizational drivers. However, most of the companies in the building sector do not have enough experience in deconstruction and reuse, which results in following other end-of-life treatment options such as demolition and recycling. Therefore, companies should take necessary actions to train the workforce to improve the productivity of their deconstruction activities and increase the reusability of the recovered building components. As discussed earlier, one possible driver to encourage companies to change their business model is the availability of regulatory incentives. However, further research should be performed to analyse the driving forces, which would help companies to integrate circularity in their business models.

The technical barriers can be categorized into deconstruction level, performance level and health and safety level. As observed in the reviewed literature, at the deconstruction level, the biggest challenge to recover building components is that the buildings are not designed for deconstruction. Although innovative design techniques can address this barrier in new buildings, it remains a significant challenge in the deconstruction of the existing built stock. At the performance level, one of the barriers to the reuse of building components after recovery is the reusability of the element (due to damages, availability of information, design challenges, etc.). According to the definition of reuse, the reusability can be defined as the extent to which the recovered building component in its new life could perform similarly to its earlier life. It is because most of the existing buildings are not designed for deconstruction, details about the existing buildings are unavailable, and proper guidelines and skills for effective deconstruction do not exist. As mentioned earlier, deconstruction can increase the reuse rate; however, there is no available guideline to help the practitioners to estimate the reuse potential of the building components before deconstruction. Therefore, further research to develop cheap and reliable techniques to investigate the reusability of building components is necessary. Moreover, while the DfD is identified as a solution to the end-of-life treatment of buildings, this design method is based on new building components. Hence, further research should be conducted to integrate the recovered building components into this design technique. At the health and safety level, as observed in Table 4, there is a strong correlation between cost and health and safety requirements of a project with deconstruction and reuse. It indicates that the increased health and safety precautions necessary for deconstruction and reuse activities (as the result of the presence of hazardous materials, etc.) could potentially increase the overall cost of the project.

## Conclusion

This study has contributed to identifying, categorizing and prioritizing the factors affecting the reuse of the components of the superstructure of a building at its end-of-life through a systematic literature review. In this study, a three-stage systematic review targeting peer-reviewed journal articles was employed. After choosing proper search words and identifying, screening and checking for eligibility, 76 journal articles were identified and reviewed thoroughly. These papers are derived from top-tier construction journals and represent the state-of-art in the body of knowledge on this topic. After identifying the reuse barriers and drivers in these articles, we categorized them based on the identified themes. Then, through the development of a correlation matrix, we tried to understand the potential interdependencies between the barriers and sought the possibility of prioritizing them. The results of this study can be used as a guideline by researchers and stakeholders in the building sector to take progressive steps towards the circularity of materials in this sector.

According to the reviewed articles, the reuse of building components is a sustainable approach that can reduce the environmental footprints of the buildings considerably. However, various obstacles hinder reuse. In this study, the challenges facing the building sector in integrating reused components in the superstructure of new buildings were uncovered, while the advantages of BCR were highlighted. Consequently, the study presented actions necessary to be taken, which could promote BCR in the building sector and recognized future research areas to address the identified gaps in the literature.

An initial look at the barriers revealed that addressing the economic factors playing a significant role in the successful implementation of reuse in the building sector, followed by technical, social, regulatory and organizational barriers. After analysing the inter-relationship between the sub-categories of barriers, it was observed that while addressing reuse obstacles requires a holistic approach, actions to overcome the social, economic and regulatory barriers should be prioritized.

In contrast to the mentioned contributions, this study has some limitations. The most important limitation of this study is its focus on the reuse of components in the superstructure of buildings and the fact that the observations may not be generalized to the substructure of buildings and the overall construction sector. Therefore, it is advised that such an investigation in other sub-divisions of the construction industry, such as foundations, roads, bridges and infrastructures, should be performed. Moreover, this paper is limited to top-tier peer-reviewed journal articles in the building sector. Hence, the correlations observed in Table 4 should be considered with caution.

## Notes

1. This term indicates that there are other references identifying the same factor.
